# Advances in Immunology of Neglected Tropical Diseases: New Control Tools and Prospects for Disease Elimination

**DOI:** 10.1155/2020/9361518

**Published:** 2020-03-16

**Authors:** Barbara Castro-Pimentel Figueiredo, Carina Silva Pinheiro, Fabio Vitarelli Marinho, Nestor Adrian Guerrero

**Affiliations:** ^1^Departamento de Bioquimica e Biofisica, Universidade Federal da Bahia, Salvador, BA, Brazil; ^2^Departamento de Biointeracao, Universidade Federal da Bahia, Salvador, BA, Brazil; ^3^Departamento de Bioquimica e Imunologia, Universidade Federal de Minas Gerais, Belo Horizonte, MG, Brazil; ^4^Centro de Biologia Molecular Severo Ochoa, Universidad Autonoma de Madrid, Madrid, Spain

Neglected tropical diseases (NTD) comprise a group of infectious viral, bacterial, and parasitic diseases with high endemicity in tropical and subtropical regions, disproportionately affecting the developing countries [1]. The WHO currently classifies 20 diseases and conditions as NTDs. The great diversity of parasites comprising NTDs hinders the development of strategies of management and the treatment of these diseases. Besides that, another barrier to overcome is the poverty and the lack of health care, sanitation, and clean water in endemic countries [2]. Due to inefficient diagnosis and also inadequate access to drugs, most of NTD infections are left untreated, which has catastrophic consequences, triggering high rates of disease spread, disability, and even death [3, 4]. It is estimated that more than one billion people worldwide are affected by NTD in 149 undeveloped countries [1]. The research on this field is also challenged by the socioeconomic, cultural, demographic, and political factors, which compromises the interest of many scientists worldwide. Although they are few compared to other fields in health science, NTD researchers have made a lot of advances in disease comprehension and novel tools for prophylaxis, diagnosis, and treatment, including vector control, vaccines, diagnostic tests, and new drugs. Without these new tools, NTD control and elimination will not happen [5, 6].

The control and elimination of NTD involve both the comprehension of immune mechanisms during parasite establishment in the host and the development of better tools to improve detection and minimize transmission. Research in this field is critical to comprehend each organism that causes these diseases and to develop new treatment for them. The immune response to NTD agents was previously investigated over the last decades. The new discoveries in this field enabled NTD researchers to better understand parasite evasion, modulation of host immune responses, and the immunopathology of infections. In this special issue, we present two manuscripts providing new insights on these topics. Quental and colleagues evaluate puerperal colostrum, discover that maternal Zika infection increases colostrum viscosity, and also modify cytokine concentrations. The altered levels of cytokines are possibly what changes colostrum viscosity, which represents a possible mechanism of adaptation of breastfeeding against Zika virus. In another original research, Duan and colleagues find that CCAAT/enhancer-binding homologous protein (CHOP), a transcriptional regulator induced by endoplasmic reticulum stress, plays a critical role in liver pathology associated with *S. japonicum* infection. Their results suggest that this protein promotes the progression of liver fibrosis, through alternative activation of macrophages. Immunological research is also important in the development of clinical tools available to address such diseases: vaccines, diagnostics, and drugs. In this issue, Bernardes and colleagues intend to investigate an antigen of *S. mansoni* as a target for vaccine development. This manuscript relates a complete analysis concerning humoral and cellular responses investigation and also the worm burden after vaccination and infection in murine model. Regarding the diagnostic based on immunological tools, Takeuchi and colleagues measure serum total and specific anti-*Ascaris* IgE to evaluate wheezing in children. They cluster these kids and investigate the relationship between wheezing and *Ascaris* infection. When it comes to leishmaniasis, Aleka and colleagues monitored visceral leishmaniasis treatment efficacy by measuring the production of cytokines before, during, and at the end of treatment. Their work presents a unique cohort study using a novel methodology to discriminate active from cured leishmaniasis patients.

In summary, this current special issue publishes five original research articles on recent findings about NTD immunology. The few number of papers published in this special edition reflects the major difficulties among NTD research and also the limited interest of the scientific community to this topic. Although the content of this special issue only covers a few topics, it reports the newest discoveries on the field and provides new data that enhance the knowledge in the field and stimulate researchers to keep investigating the NTD in the future. These diseases take many lives yearly and challenge many researches around the world, so the works published here enhance the understanding of NTDs and help experts in this inspiring field.

*Barbara Castro-Pimentel Figueiredo* *Carina Silva Pinheiro* *Fabio Vitarelli Marinho* *Nestor Adrian Guerrero*

## References

[1] World Health Organization (2020). Neglected tropical diseases. https://www.who.int/neglected_diseases/diseases/en/.

[2] Hotez P. J., Aksoy S., Brindley P. J., Kamhawi S. (2020). World neglected tropical diseases day. *PLoS Neglected Tropical Diseases*.

[3] Molyneux D. H., Savioli L., Engels D. (2017). Neglected tropical diseases: progress towards addressing the chronic pandemic. *The Lancet*.

[4] Nii-Trebi N. I. (2017). Emerging and neglected infectious diseases: insights, advances, and challenges. *BioMed Research International*.

[5] Hotez P. J., Pecoul B., Rijal S. (2016). Eliminating the neglected tropical diseases: translational science and new technologies. *PLoS Neglected Tropical Diseases*.

[6] Ortu G., Williams O. (2017). Neglected tropical diseases: exploring long term practical approaches to achieve sustainable disease elimination and beyond. *Infectious Diseases of Poverty*.

