# Corrigendum to “Advances in Immunology of Neglected Tropical Diseases: New Control Tools and Prospects for Disease Elimination”

**DOI:** 10.1155/2020/1948240

**Published:** 2020-10-16

**Authors:** Barbara Castro-Pimentel Figueiredo, Carina Silva Pinheiro, Fabio Vitarelli Marinho, Nestor Adrian Guerrero

**Affiliations:** ^1^Departamento de Bioquimica e Biofisica, Universidade Federal da Bahia, Salvador, BA, Brazil; ^2^Departamento de Biointeracao, Universidade Federal da Bahia, Salvador, BA, Brazil; ^3^Departamento de Bioquimica e Imunologia, Universidade Federal de Minas Gerais, Belo Horizonte, MG, Brazil

In the article titled “Advances in Immunology of Neglected Tropical Diseases: New Control Tools and Prospects for Disease Elimination” [1], author Nestor Adrian Guerrero was affiliated to Centro de Biologia Molecular Severo Ochoa, Universidad Autonoma de Madrid, Madrid, Spain, which is incorrect. The correct affiliation for this author is Departamento de Biointeracao, Universidade Federal da Bahia, Salvador, BA, Brazil.

The corrected list of affiliations is shown in the author information above.
